# Rising trend and regional disparities of the global burden of disease attributable to ambient low temperature, 1990-2019: An analysis of data from the Global Burden of Disease 2019 study

**DOI:** 10.7189/jogh.14.04017

**Published:** 2024-04-19

**Authors:** Jiangdong Liu, Mengmeng Li, Zhou Yang, Di Liu, Ting Xiao, Jian Cheng, Hong Su, Chun-Quan Ou, Jun Yang

**Affiliations:** 1 Department of Environmental Health, School of Public Health, Key Lab of Public Health Safety of the Ministry of Education, NHC Key Lab of Health Technology Assessment, IRDR ICoE on Risk Interconnectivity and Governance on Weather/Climate Extremes Impact and Public Health, Fudan University, Shanghai, China; 2 State Key Laboratory of Organ Failure Research, Department of Biostatistics, School of Public Health, Southern Medical University, Guangzhou, China; 3 State Key Laboratory of Oncology in South China, Guangdong Provincial Clinical Research Center for Cancer, Sun Yat-sen University Cancer Center, Guangzhou, China; 4 The Key Laboratory of Advanced Interdisciplinary Studies, The First Affiliated Hospital of Guangzhou Medical University, Guangzhou, China; 5 School of Public Health, Guangzhou Medical University, Guangzhou, China; 6 Department of Epidemiology and Biostatistics, School of Public Health, Anhui Medical University, Hefei, China

## Abstract

**Background:**

Previous studies on the effect of global warming on the global burden of disease have mainly focussed on the impact of high temperatures, thereby providing limited evidence of the effect of lower temperatures.

**Methods:**

We adopted a three-stage analysis approach using data from the Global Burden of Disease 2019 study. First, we explored the global burden of disease attributable to low temperatures, examining variations by gender, age, cause, region, and country. Second, we analysed temporal trends in low-temperature-related disease burdens from 1990 to 2019 by meta-regression. Finally, we fitted a mixed-effects meta-regression model to explore the effect modification of country-level characteristics.

**Results:**

In 2019, low temperatures were responsible for 2.92% of global deaths and 1.03% of disability-adjusted life years (DALYs), corresponding to a death rate of 21.36 (95% uncertainty interval (UI) = 18.26, 24.73) and a DALY rate of 335 (95% UI = 280, 399) per 100 000 population. Most of the deaths (85.12%) and DALYs (94.38%) attributable to low temperatures were associated with ischaemic heart disease, stroke, and chronic obstructive pulmonary disease. In the last three decades, we observed an upward trend for the annual number of attributable deaths (*P* < 0.001) and a downward trend for the rates of death (*P* < 0.001) and DALYs (*P* < 0.001). The disease burden associated with low temperatures varied considerably among regions and countries, with higher burdens observed in regions with middle or high-middle socio-demographic indices, as well as countries with higher gross domestic product per capita and a larger proportion of ageing population.

**Conclusions:**

Our findings emphasise the significance of raising public awareness and prioritising policies to protect global population health from the adverse effects of low temperatures, even in the face of global warming. Particular efforts should be targeted towards individuals with underlying diseases (e.g. cardiovascular diseases) and vulnerable countries or regions (e.g. Central Asia and central Europe).

Climate change has become a prominent public health concern in the 21st century, attracting significant attention from the research community. When considering global warming, existing epidemiological studies have primarily focussed on estimating and predicting the disease burden associated with heat [1-4], partially neglecting the impact of low temperatures. However, evidence indicates that cold weather imposes a greater disease burden than hot weather [5-8]. For instance, in the analysis of the global disease burden for all causes in 2019, low temperatures ranked sixth among 38 risk factors, while high temperatures ranked 26th [9].

There is currently a need for comprehensive and reliable assessments of the global cold-related disease burden to inform actions aimed at addressing the effect of low temperatures. However, most studies in this field have been limited to specific cities or countries [8,10,11], leading to challenges in comparability due to differences in their population characteristics, study designs, and statistical methods. Furthermore, only a few studies have explored temporal trends in cold-related mortality over the past few decades, leaving a gap in our understanding of the effects of cold temperatures within the context of global warming [12,13]. Additionally, gaining insights into the geographical heterogeneity and health inequality associated with cold-related health effects could further help with mitigating the detrimental impacts of low temperatures [14]. While some research has investigated the potential influence of local factors on the relationship between cold and health outcomes [15,16], the findings have shown significant disparities, probably attributed to variations in population and socioeconomic characteristics. This leaves a need for cross-national investigation that explores how demographic and socioeconomic factors influence the impact of low temperatures at the national level.

The Global Burden of Disease (GBD) 2019 study has estimated for the first time the disease burden attributable to low temperatures in regions with various climatic conditions, socioeconomic statuses, and demographic characteristics from 1990 to 2019; however, it solely provided overall estimates and did not delineate possible temporal and spatial variations in this burden.

In this study, which we based on data from the GBD 2019 study, we set three objectives: To provide a comprehensive overview and examination of the global disease burden caused by low temperatures while accounting for factors such as age, gender, region, country, and disease type; to investigate the changes in disease burden attributed to low temperatures over the period from 1990 to 2019; and to explore the extent to which country-level demographic and socioeconomic characteristics affect the disease burden of low temperatures.

## METHODS

### Brief overview of the GBD study

The GBD 2109 study quantified the health effects of risk factors on hundreds of diseases and injuries by age, gender, and geographic region for 204 countries and territories categorised into 21 regions and 7 super regions [17]. It was the first study of its kind to identify low temperature exposure as a global risk factor. Furthermore, the GBD 2019 study used the socio-demographic index (SDI), a composite indicator of income per capita, average years of schooling, and fertility rate in females younger than 25 years, to divide countries and territories into five SDI levels. Additionally, the study divided all causes of health losses related to low temperatures into a hierarchical structure consisting of four nested levels, increasing in specificity from level 1 to level 4 (Figure S1 in the **Online Supplementary Document**). More details on the GBD study are available on its website [18].

### Estimation of disease burden related to low temperatures in GBD Study 2019

The GBD 2019 study estimated the disease burden attributable to low temperatures through a well-established estimation process [9,19], presenting relevant indicators as point estimates with their corresponding 95% uncertainty intervals (UIs) [20]. Briefly, it collected cause-specific mortality data at the county or municipality level, which were linked to local daily temperature estimates from the ERA5 data set with high spatial and temporal resolutions [21]. Then, the study team used a robust meta-regression framework, the meta-regression–Bayesian regularised trimmed tool, to estimate temperature-mortality curves. This approach not only identified temperature-related causes of death by estimating risk outcome scores, but also effectively handled heterogeneous data by pooling the relative risks (RRs) across different temperature zones [20]. Specifically, only causes with positive risk outcome scores were included in subsequent analyses. Meanwhile, the theoretical minimum risk exposure level for temperature (TMREL) was calculated for each location and year, defining low temperature as values below the TMREL. Subsequently, the GBD 2019 team calculated population attributable fractions (PAFs) using spatially and temporally varying TMREL and estimated RRs, and then used these values to estimate cause-specific disease burden attributable to low temperatures (Method S1 in the **Online Supplementary Document**) [9,19,20].

In this study, we used the number and rates of deaths and disability-adjusted life years (DALYs) per 100 000 population as the primary indicators for assessing the burden of cold-related diseases. Additionally, we used age-standardised rates of death and DALY, calculated using the global population for age standardisation, as supplementary indicators to account for differences in population age composition when comparing disease burdens across countries and territories. Moreover, we mainly used hierarchical information on cold-related disease from level 1 to level 3.

### Data on socioeconomic characteristics

To identify potential modifiers that influence the disease burden attributed to low temperatures, we collated a variety of country-level indicators from the World Bank databases [22] for the 204 countries and territories, which we then categorised into four groups:

1. Demographic characteristics: life expectancy at birth in years, population density, the proportion of female, and the proportion of population aged ≥65 years;
2. Socioeconomic conditions: Gross domestic product (GDP) per capita, health expenditure per capita, physicians availability per 1000 people,
3. Urban characteristics: Proportion of urban population and annual urban population growth (%);
4. Air pollutants: Annual concentrations of fine particulate matter (PM_2.5_).

Given that most of the above-mentioned data were exclusively accessible for the period spanning from 2000 to 2018, we delimited the temporal scope of this section to the same time frame, i.e. from 2000 to 2018.

### Statistical analysis

We first analysed the disease burden associated with low temperatures by country, age, gender, and SDI level using attributable deaths and DALYs along with their corresponding rates or age-standardised rates with 95% UIs. Second, we used meta-regression to investigate the temporal variation in the disease burden attributable to cold during 1990–2019, using time as the independent variable and the disease burden outcomes as the dependent variables. This method incorporates the information provided by point estimates and account for the uncertainties (sampling errors) associated with the point estimates by assigning weights [23], thus producing a more precise and reliable temporal trend in the disease burden. The formula for the meta-regression model is as follows:

*θ̂_t_* = θ + *βx_t_* + *ε_t_* + *ζ_t_* *t* = 1, …, 30

where *θ̂_t_* is the cold-related disease burden in year *t*; *θ* is the intercept; *x_t_* is the year *t*; *β* is the average annual change in cold-related disease burden over the last 30 years; and *ε_t_* and *ζ_t_* are the sampling error through which the effect size of a disease burden deviates from its true effect and heterogeneity variance, respectively. The equation includes both fixed effects (the coefficient *β*) and random effects (*ζ*_t_). When *β* is statistically significant, it indicates a significant increasing (*β*>0) or decreasing trend (*β*<0) in the disease burden; otherwise, no significant trend is observed. Lastly, we explored the effects of country-level indicators on cold-related disease burden using an extended mixed-effects meta-regression model [24], which has advantages in comprehensively capturing the complexity of effect sizes by effectively accounting for the inherent variability within and between countries, while simultaneously considering the uncertainties associated with secondary data. By employing a meta-analysis framework, we integrated both interval estimates and point estimates from the GBD 2019 data. Additionally, the model incorporated a country-specific random intercept to account for within-country correlations, while the potential modified factors were included as fixed-effect terms. Coefficients and the 95% confidence interval (CI) of country-level indicators were then calculated (Method S2 in the **Online Supplementary Document**).

We conducted all statistical analyses in R, version 4.1.0 (R Core Team, Vienna, Austria) with the ‘mixmeta’ package [24]. A two-tailed *P*-value <0.05 denoted statistical significance.

## RESULTS

### Cold-related disease burden by subgroup in 2019

The GBD 2019 study estimated that low temperatures accounted for 2.92% and 1.03% of global deaths and DALYs in 2019, equivalent to a death rate of 21.36 (95% UI = 18.26, 24.73) and DALY rate of 335 (95% UI = 280, 399) per 100 000 population, which were 5.37 times and 2.2 times the estimates of burdens attributable to high temperatures (Table S1 in the **Online Supplementary Document**).

**Figure 1:**
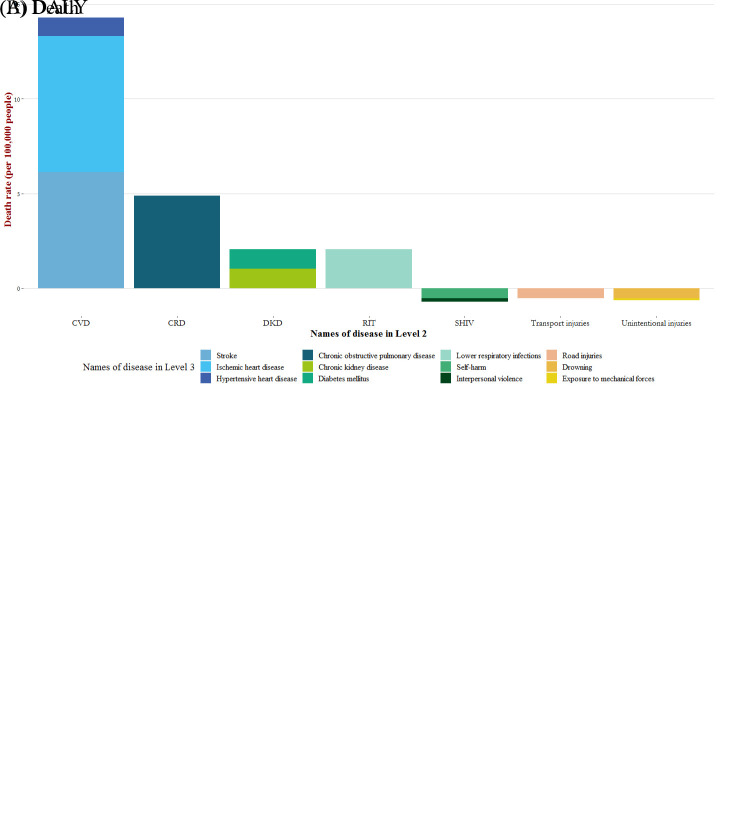
Global disease burden attributable to low temperature stratified by cause in GBD 2019. The diseases were grouped by cause at level 2 and level 3, respectively. CMNND – communicable, maternal, neonatal, and nutritional diseases, CRD – chronic respiratory diseases, CVD – cardiovascular diseases, DKD – diabetes and kidney diseases, RIT – respiratory infections and tuberculosis, SHIV – self-harm and interpersonal violence

When considering gender disparities, men experienced 1.38 times higher age-standardised death rates and 1.35 times higher DALY rates attributed to cold compared to women. Regarding age groups, both age-standardised death and DALY rates related to low temperatures showed a J-shaped trend (Figure S2 in the **Online Supplementary Document**), with a significant increase in the elderly population (≥70 years). In terms of disease causes, most of the total number of deaths and DALYs attributable to cold were from non-communicable diseases (NCDs). The top three causes of cold-related death and DALY rates per 100 000 population in GBD level 3 diseases were ischaemic heart disease, stroke, and chronic obstructive pulmonary disease (**Figure 1**; Table S2 − 3 in the **Online Supplementary Document**).

### Spatial patterns in cold-related disease burden in 2019

**Figure 2:**
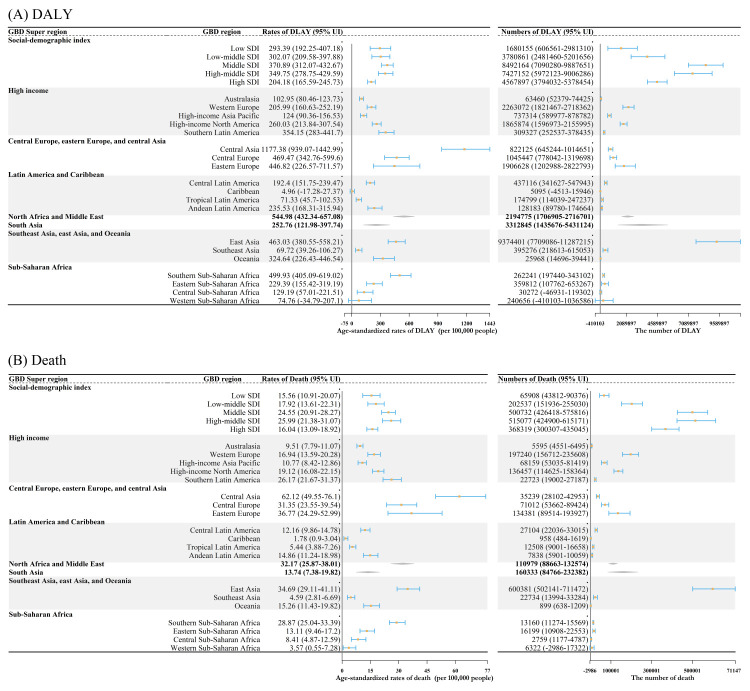
Disease burden attributable to low temperature in different GBD super regions. **Panel A.** Number and age-standardised rate of DALYs attributable to low temperatures in 21 geographical and 5 SDI level regions. **Panel B.** Number and age-standardised rate of deaths attributable to low temperatures in 21 geographical and 5 SDI level regions. DALY – disability-adjusted life-year, SDI – socio-demographic index.

The burden of disease attributable to low temperatures significantly varied across GBD regions worldwide (**Figure 2**). In 2019, East Asia had the highest number of low-temperatures-related deaths (0.60 million; 95% UI = 0.50, 0.70), followed by Western Europe, South Asia, and High-income North America. Meanwhile, East Asia had the highest number of DALYs (9.37 million; 95% UI = 7.71, 11.29), followed by South Asia, Western Europe, and North Africa and Middle East. Corresponding age-standardised death rates per 100 000 population ranged from 1.78 (95% UI = 0.90, 3.04) in the Caribbean to 62.12 (95% UI = 49.55, 76.10) in Central Asia, while age-standardised DALY rates per 100 000 population ranged from 4.96 (95% UI = −17.28, 27.37) in Southeast Asia to 1177 (95% UI = 939, 1443) in Central Asia. Moreover, cardiovascular diseases (CVDs), particularly ischaemic heart disease, were the found to be the predominant factors contributing to the burden associated with low temperatures demonstrated across different regions.

**Figure 3:**
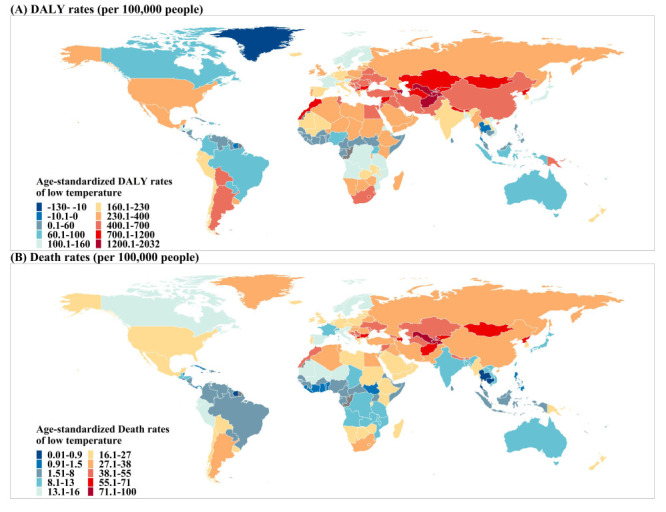
Age-standardised rate per 100 000 population of low temperature-related DALYs and deaths. **Panel A.** DALYs. **Panel B.** Deaths. DALYs – disability-adjusted life-years.

We also observed substantial differences in the burden of disease attributable to cold among countries (**Figure 3**; Figure S3 in the **Online Supplementary Document**). In 2019, China had the highest number (in millions) of cold-related deaths (0.58; 95% UI = 0.49, 0.69) and DALYs (9.05; 95% UI = 7.40, 10.93), followed by the United States, India, and Russia. The top three countries with the highest estimated age-standardised death rates per 100 000 population due to low temperatures were Uzbekistan (96.37; 95% UI = 70.4, 123.13), Lesotho (91.58; 95% UI = 67.61, 118.65), and Tajikistan (91.2; 95% UI = 56.46, 130.4). Additionally, Lesotho had the highest age-standardised rate of DALYs attributable to low temperatures per 100 000 population (2031; 95% UI = 1472, 2743), followed by Uzbekistan, Tajikistan, and Afghanistan.

### The 30-year temporal trends in the cold-related disease burden.

**Figure 4:**
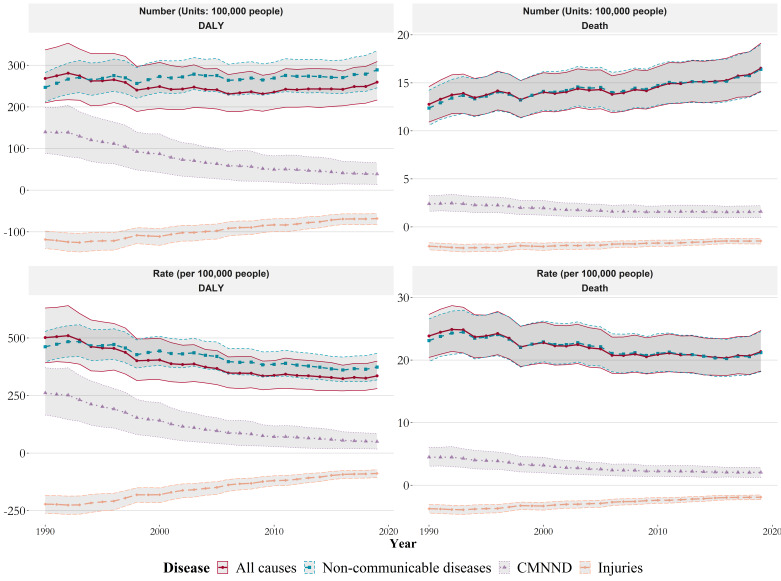
Temporal trends in the burden of all-cause and level 1 cause-specific diseases attributable to low temperatures from 1990 to 2019 at the global level. We grouped the diseases by cause at level 1. The burden was indicated by the number and age-standardised rate of DALYs and deaths attributable to low temperature. The shaded area corresponding to each line represented the 95% UIs. CMNND – communicable, maternal, neonatal, and nutritional diseases, DALY – disability-adjusted life-year, UI – uncertainty intervals.

We explored the temporal trends of all-cause and cause-specific (level 1) disease burden attributable to low temperatures from 1990 to 2019 globally (**Figure 4**). During this period, the annual number of deaths (in millions) due to low temperatures increased from 1.28 (95% UI = 1.09, 1.46) to 1.65 (95% UI = 1.41, 1.91), indicating an overall upward trend (*P* < 0.001). However, the corresponding annual attributable death rate per 100 000 population showed an overall downward trend (β = −0.15; *P* < 0.001), decreasing from 23.86 (95% UI = 20.43, 27.31) to 21.36 (95% UI = 18.26, 24.73). Moreover, while the annual number of attributable DALYs showed no statistically significant trend (*P* > 0.05), the attributable DALYs rate per 100 000 population had a decreasing trend (β = −5.62; *P* < 0.001). Furthermore, we observed inconsistent temporal trends in cause-specific disease burden attributable to low. Globally, there was a notable decrease in the prevalence of communicable, maternal, neonatal, and nutritional diseases related to cold temperatures. Conversely, NCDs had a decreasing trend in death and DALY rates, but the number of deaths increased, while DALYs showed no significant variation.

**Figure 5:**
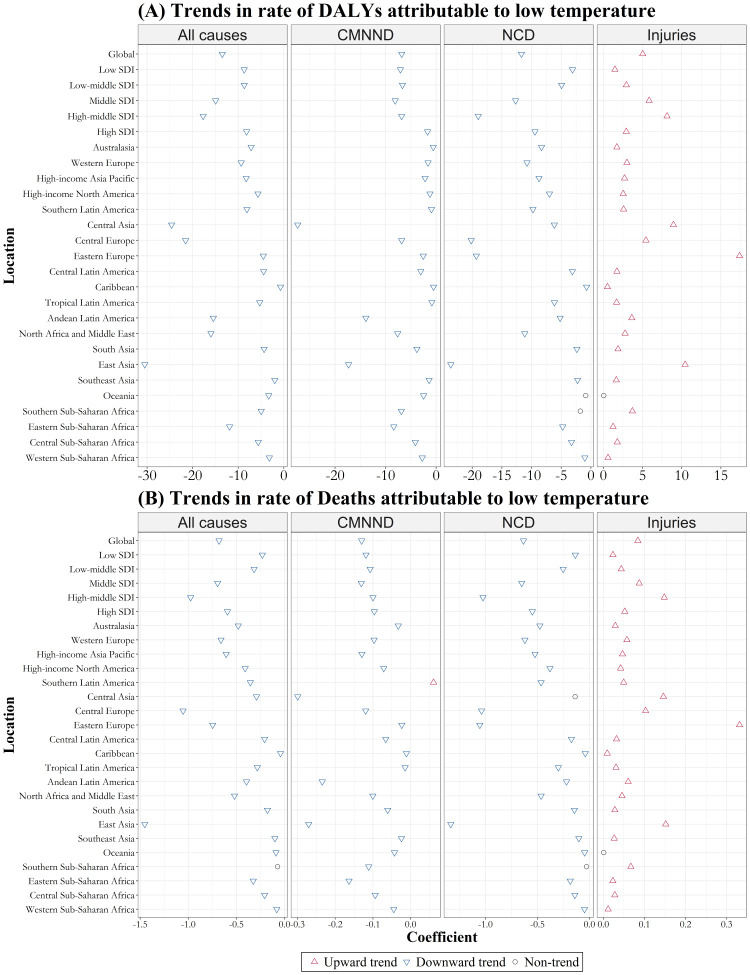
Temporal trends in age-standardised rate of DALYs and deaths attributable to low temperatures from 1990 to 2019 among 21 geographical and five SDI level regions. **Panel A.** DALYs. **Panel B.** Deaths. CMNND – communicable, maternal, neonatal, and nutritional diseases, DALYs – disability-adjusted life-years, NCD – non-communicable diseases.

Additionally, the temporal trends in disease burden attributed to cold varied regionally (**Figure 5**; Table S4 and Figures S4–5 in the **Online Supplementary Document**). Specifically, among the 21 GBD regions, the annual number of deaths caused by low temperatures showed an increasing trend in 13 regions and no discernible trend in 8 regions, with no region displaying a downward trend. However, the corresponding annual death rates related to low temperatures declined over the past 30 years in all except for four specific regions where no significant declining trend was observed. Meanwhile, the trends in disease burden due to low temperatures also varied by country. We found the most substantial annual increases in age-standardised DALY rates and death rates per 100 000 population due to low temperatures in Lesotho (β = 17.39) and Uzbekistan (β = 1.34) (*P* < 0.001). Moreover, Mongolia (β = −69.27; *P* < 0.001) and China (β = −1.48; *P* < 0.001) had the most significant annual decreases in attributable DALY rates and death rates, respectively.

### The effect of SDI on disease burden related to low temperatures

Among the five SDI levels in 2019 (**Figure 2**), the middle SDI group showed the highest number of low-temperature-related DALYs in millions (8.5; 95% UI = 7.1, 9.9) and the highest corresponding age-standardised DALY rate per 100 000 population (371; 95% UI = 312, 433). The middle-high SDI group had the highest number of cold-related deaths (25.99; 95% UI = 21.38, 31.07) and the highest corresponding age-standardised rates (0.52; 95% UI = 0.42, 0.62). The disease burden attributable to low temperature showed a nonlinear trend in the five SDI regions, with similar nonlinear association appearing at the country level (Figure S6 in the **Online Supplementary Document**). From 1990 to 2019, age-standardised DALY rates and death rates showed a decreasing trend across the five SDI regions, but the number of DALY and deaths increased in all SDI regions except for DALYs in the middle SDI region and deaths in the high SDI region (**Figure 5**).

### Effect of country-level indicators on the cold-related disease burden

Regarding demographic indicators, countries with a lower proportion of women and a higher proportion of older people demonstrated a greater disease burden associated with low temperatures. Conversely, countries with longer life expectancy had a lower burden of cold-related diseases. Regarding economic factors, we found that a higher GDP per capita contributed to an increased burden of cold-related diseases. Conversely, the burden decreased significantly with a higher health expenditure per capita. Among urban characteristics indexes, only the urban population was positively correlated with cold-related DALY rates (**Table 1**).

**Table 1:** Associations between the country-level indicators and low temperature-related disease burdens based on univariate extended random-effects meta-regression

Indicator	DALY rates, β (95% CI)	*P*-value	Death rates, β (95% CI)	*P*-value
**Demographic indicators**				
Life expectancy at birth in years	−10.85 (−12.42, −9.28)	<0.001	−0.13 (−0.25, −0.01)	<0.05
Population density in people per km^2^	0.02 (−0.02, 0.05)	>0.05	−0.001 (−0.003, 0.002)	>0.05
Proportion of women in total population	−5.06 (−6.59, −3.61)	<0.001	−0.30 (−0.55, −0.06)	<0.05
Proportion of population aged ≥65 years in total population	11.13 (9.38, 12.86)	<0.001	0.37 (0.16, 0.59)	<0.001
**Socioeconomic indicators**				
GDP per capita in USD 1000	0.42 (0.09, 0.74)	<0.05	0.002 (−0.07, 0.07)	>0.05
Health expenditure per capita in USD 1000	−14.30 (−18.30, −10.30)	<0.001	−0.90 (−1.70, −0.20)	<0.05
Physicians per 1000 people	−2.86 (−4.50, −0.87)	<0.01	−0.01 (−0.35, 0.34)	>0.05
**Urban characteristics**				
Proportion of urban population in total population	2.84 (1.70, 3.98)	<0.001	0.07 (−0.02, 0.17)	>0.05
Annual percentage of urban population growth	−0.42 (−1.26, 0.42)	>0.05	−0.06 (−0.21, −0.09)	>0.05
**Air pollution**				
PM_2.5_ in μg/m^3^	−0.09 (−0.33, 0.14)	>0.05	−0.01 (−0.05, 0.02)	>0.05

β – correlation coefficient, CI – confidence interval, DALY –disability-adjusted life years

## DISCUSSION

From a global perspective, this is the first comprehensive and systematic analysis of spatiotemporal patterns in the cold-related burden of various diseases in the past three decades. It is also the first study of factors that potentially affect the burden of disease attributable to low temperatures at the country level. Globally, low temperatures were responsible for 2.92% of death rates and 1.03% of DALY rates in 2019. The disease burden related to low temperatures showed substantial variations across different regions and countries. Although the overall number of deaths associated with low temperatures has been increasing over the past 30 years, the rates of deaths and DALYs displayed contrasting trends. Furthermore, in view of country-level indicators, we identified a higher disease burden in economically developed and ageing countries.

The assessment of different susceptible diseases across regions is important for informing local public health policy and the future allocation of infrastructure resources. Our findings highlight the global dominance of CVDs, particularly ischaemic heart disease, in the burden associated with low temperatures, aligning with previous research [10,25]. Prominently, despite significant regional variations in the burden of ischaemic heart disease attributable to low temperatures, it remained the primary contributor to the disease burden associated with low temperatures in almost all regions. Meanwhile, attention should also be given to respiratory diseases, accounting for 37.7% of the total DALYs and 32.4% of deaths attributed to low temperature worldwide. The underlying mechanism for these findings may involve the constriction of peripheral blood vessels and increased cardiac afterload in patients with pre-existing cardiovascular conditions due to low temperatures [26]. Additionally, cold weather might reduce the immunity of the mucous membranes of the respiratory tract, indirectly increasing the risk of infection. Moreover, regions with middle or high-middle SDI exhibited a heavier cold-related burden, which might be attributed to a higher prevalence of CVDs in these areas [9].

The development of effective measures against low temperatures requires an accurate grasp of temporal trends in the burden of cold-attributable diseases. Currently, there are still great inconsistencies in the trends of the disease burden related to cold [12,27,28], which may be due to varying policies, economic levels, and population characteristics in different regions. From a global perspective, we found that even under the context of global warming, the number of deaths attributable to low temperature had increased in the past 30 years, although the rate of deaths showed a slight decrease after adjusting for population size, with further adjustments for age structure leading to a more pronounced decline. We also observed similar trends for DALYs. These results suggest that, in the context of an ageing and growing population, the decline in cold-related disease burden due to global warming did not occur as expected. Additionally, the significant reduction in the burden of communicable, maternal, neonatal, and nutritional diseases related to cold may be attributed to improvements in living conditions and medical care in recent years. Meanwhile, the observed regional variations in trends of cold-related disease burden indicate that the cold adaptation has a complex geographic pattern. However, previous studies have presented uncertainties regarding whether the declining trends in overall disease burden caused by cold conditions are attributed to global-level adaptation to cold or non-climatic factors, such as advancements in health care and infrastructure [13,29,30]. Further in-depth research from multidisciplinary perspectives, including geography, sociology, and meteorology, is necessary to elucidate the mechanisms driving the changes in cold-related mortality.

Previous studies exploring the socioeconomic factors related to low temperatures reported discrepant results [15,16]. These disparities may stem from diverse factors, including population heterogeneity, methodological discrepancies, and variations in sample sizes. Therefore, by uniformly analysing data from 204 regions and territories over a span of 19 years, we observed that countries with higher GDP per capita had a higher cold-related disease burden. Several factors could contribute to this phenomenon. First, countries with higher GDP per capita, primarily located in Europe and North America, have higher latitudes and lower annual temperatures compared to other regions and therefore experience a larger burden of cold-related diseases [20]. Additionally, the health care system structure emerges as another potential explanatory factor. The health care systems of countries with higher GDP per capita generally have a higher proportion of private health care, resulting in greater health inequities [31]. For instance, the USA, which has a higher proportion of private health care expenditures in relation to the total health spending [32], showed a considerably higher cold-related age-standardised DALY rate (289 per 100 000 population) compared to its neighbouring country, Canada (89 per 100 000 population), despite having a higher GDP. Furthermore, the underlying burden of disease due to low temperatures might also contribute to the result. Cold-related burdens predominantly arise from CVDs [20], which exhibit higher mortality rates in high-income compared to low-income regions, as shown by the GBD study 2019. Moreover, consistent with previous studies [8,33], we observed that the elderly may exhibit a higher vulnerability to low temperatures, which can be attributed to the increased prevalence of chronic cardiopulmonary diseases among this population.

Our study has some limitations. First, due to a lack of data, we were unable to analyse the effects of some country-level indicators such as hospital beds per thousand population and circulating pathogens such as flu, which may be associated with cold vulnerability [15,34]. Second, while the GBD 2019 study accounted for spatial heterogeneity between temperature zones, it did not explicitly model factors such as infrastructure and behaviour characteristics. Third, the GBD database itself has limitations, such as the quality of the data collected in different countries, as reported elsewhere [35,36]. Moreover, cold spells (meaning extreme temperature events) can also directly affect the human body and the economies of affected countries. Here we were unable to analyse their impact on the global level. Lastly, the burden of low temperatures estimated by GBD 2019 only considered the very short lag effects of low temperatures, which can actually last up to three weeks or more, thereby resulting in a possible underestimation of the global burden of disease attributable to low temperatures.

## CONCLUSIONS

Our study provides evidence that low temperatures exert a substantial global disease burden, highlighting an urgent need for proactive interventions by health policymakers. Moreover, our results help determine which countries or regions, populations, and specific diseases might be especially vulnerable in this regard, as well as which country-level modifiers may influence cold susceptibility at a global scale. These findings can help with the effective allocation of resources and can inform policymakers and stakeholders to prioritise investments in measures that could decrease the impact of low temperatures.

## Additional material


Online Supplementary Document
