# Supplementary material for: Rising trend and regional disparities of the global burden of disease attributable to ambient low temperature, 1990-2019: An analysis of data from the Global Burden of Disease 2019 study
Source: J Glob Health. 2024 Apr 19;14:04017. doi: 10.7189/jogh.14.04017 (PMC11026037; doi:10.7189/jogh.14.04017)
Supplement: Online Supplementary Document [file jogh-14-04017-s001.pdf]

Supplementary materials

OUTLINE

Title	Page
**Method S1.** Estimation of disease burden related to low temperatures in GBD.	**1-2**
**Method S2.** The extended mixed-effects meta-regression model.	**3**
**Table S1.** Global burden of all causes attributable to low and high temperature in 2019.	**4**
**Table S2.** Twelve leading causes of total death rates attributable to low temperatures in 2019 by region.	**5-8**
**Table S3.** Twelve leading causes of total DALY rates attributable to low temperatures in 2019 by region.	**9-12**
**Table S4.** Global age-standardized rates of death and DALY attributable to high temperature from 1990-2019.	**13-20**
**Figure S1.** Low-temperature-related diseases studied within GBD 2019.	**21**
**Figure S2.** Age-standardized rates of deaths and DALYs attributable to low temperatures stratified by age and gender.	**22**
**Figure S3.** The number of low temperature-related deaths and DALYs across countries.	**23**
**Figure S4.** Temporal trends in numbers of DALYs and deaths attributable to low temperatures from 1990 to 2019 among 21 geographical and five SDI level regions.	**24**
**Figure S5.** Temporal trends of age-standardized DALY and death rates attributable to low temperature across countries from 1990-2019.	**25**
**Figure S6.** Correlation between SDI value and age-standardized rate of DALY and death attributable to low temperature by country.	**26**

Method S1: Estimation of disease burden related to low temperatures

As for estimating low-temperature attributable burden of disease, similar to conventional risk factors, there are mainly four steps:

(1) Determination of the inclusion of low temperatures - outcome pairs and data collection: the World Cancer Research Fund criteria for convincing or probable evidence of risk–outcome pairs were used to determine which risk-outcome pair such as low temperatures and all causes mortality are of value for analysis. When pairs are identified, data is collected for further calculations. Take low temperatures as an example. Hourly temperature estimates for each location (at the county or municipality level) were collected from ERA5, a gridded reanalysis dataset produced by the European Centre for Medium-Range Weather Forecasts with 0.25°×0.25°spatial and subdaily temporal resolutions, including uncertainty estimates on a 0.5°×0.5°spatial and three-hourly resolution, and then daily mean temperature were calculated. Individual death information was obtained from the GBD cause of death(CoD) database for vital registration data sources. After linking cause-specific mortality to daily mean temperature estimates, the GBD study modeled relative risks (RR) for different causes of death using a two-dimensional spline within a Bayesian meta-regression framework, specifically MR-BRT (meta-regression–Bayesian, regularized, trimmed) tool. This allowed the GBD study to identify causes with a positive risk-outcome score, indicating an association with temperature. Only causes with a risk-outcome score greater than zero were considered to be cold-related and were included in subsequent analyses. Therefore, seven disease causes (Level 2) were identified as outcomes related to low

temperatures (Figure S2).

(2) Estimation of the population attributable fraction (PAF): When identifying low-temperature-related causes by MR-BTR, meta-analyses of relative risks of low temperatures were also conducted to construct a function of low temperatures (exposure–response curves) based on daily mean temperature and temperature zone. Modelling along different temperature zones and integrating data from all locations into one model contributed to stabilizing the estimates across zones. Then spatiotemporal Gaussian process regression was used to estimate mean levels of exposure by age-sex-location-year, and a measure of dispersion such as standard deviation (SD) was modelled to estimate the distribution of exposure across individuals. After that, the theoretical minimum risk level (TMREL) for temperature, defined as the low point of the risk function, was estimated for each given location and year, defining low temperatures as values below TMREL. Since TMREL varies considerably with locations (e.g., higher in hot areas than colder regions), years, and diseases, spatially and temporally varying TMREL was employed to future account for regional heterogeneity. At last, PAF can be calculated by the standard formula with indicators including the exposure levels for low temperature, the RR of the outcome as a function of exposure (exposure–response curves), and counterfactual risk factor exposure (TMREL).

(3) Calculation of low temperatures attributable burden: through PAF, specific-cause attributable deaths and DALYs were estimated. The more detailed process has been presented in the website (http://www.healthdata.org/gbd/).

Method S2: The extended mixed-effects meta-regression model

The extended mixed-effects meta-regression model adopted in this study is as follow:

$$Y_{\mathrm{it}} = \beta' X_{it} + b' Z_{\mathrm{it}} + u_{it} \qquad i = 1, \cdots 204; t = 1, \cdots 19 \qquad (1)$$

where Y_{it} represents the burden of disease related to low temperatures for the country i in the year t; X_{it} represents the matrix of order $k*t$ independent variables (k refers to the number of potential modified factors such as socioeconomic characteristics indicators; β refers to fixed-effects coefficients. The random part of the model, $Z_{it}b$, indicates the deviation from the averages of outcome in terms of 19 time points and composing the random-effects matrix Z_i, with coefficients b. The vector μ_{it} defines the unit-level sampling errors.

Table S1. Global burden of all causes attributable to low and high temperature in 2019.

Measure	Location	Cause	Risk	Metric	Year	Point estimate	95% Uncertainty interval
Deaths	Global	All causes	Low temperature	Number	2019	1,652,978	(1,413,026, 1,913,431)
Deaths	Global	All causes	Low temperature	Rate	2019	21.36	(18.26, 24.73)
DALYs (Disability-Adjusted Life Years)	Global	All causes	Low temperature	Number	2019	25,954,684	(21,667,683, 30,902,494)
DALYs (Disability-Adjusted Life Years)	Global	All causes	Low temperature	Rate	2019	335.44	(280.04, 399.39)
Deaths	Global	All causes	High temperature	Number	2019	307,847	(223,025, 455,767)
Deaths	Global	All causes	High temperature	Rate	2019	3.98	(2.88, 5.89)
DALYs (Disability-Adjusted Life Years)	Global	All causes	High temperature	Number	2019	11,696,516	(8,189,011, 19,372,703)
DALYs (Disability-Adjusted Life Years)	Global	All causes	High temperature	Rate	2019	151.17	(105.84, 250.38)

Note: Rate: per 100,000 population

Table S2. Twelve leading causes of total death rates attributable to low temperatures in 2019 by region. Causes are ranked according to global estimates of age-standardized death rates (per 100,000 people) provided by the GBD Study 2019. The rates are listed and colored in each cell: Shades of blue indicate death rates less than zero whereas red indicates death rates greater than zero. (Notes: IHD=Ischemic heart disease, COPD=Chronic obstructive pulmonary disease, LRI=Lower respiratory infections, CKD=Chronic kidney disease, DM=Diabetes mellitus, HHD=Hypertensive heart disease, EMF=Exposure to mechanical forces, IV=Interpersonal violence, RI=Road injuries.)

Region	***1***	***2***	***3***	***4***	***5***	***6***	***7***	***8***	***9***	***10***	***11***	***12***
**Global**	IHD 7.23	Stroke 6.16	COPD 4.94	LRI 2.02	CKD 1.05	DM 0.99	HHD 0.99	EMF -0.12	IV -0.19	Self-harm -0.50	RI -0.52	Drowning -0.52
**Social-demographic index**												
Low SDI	IHD 5.51	COPD 3.98	Stroke 3.07	LRI 1.50	HHD 1.09	CKD 0.93	DM 0.87	EMF -0.09	IV -0.21	Drowning -0.32	Self-harm -0.34	RI -0.44
Low-middle SDI	IHD 5.82	COPD 5.76	Stroke 4.30	LRI 1.30	CKD 0.82	DM 0.79	HHD 0.78	EMF -0.08	IV -0.17	Self-harm -0.43	Drowning -0.43	RI -0.54
Middle SDI	IHD 7.86	Stroke 7.18	COPD 6.38	HHD 1.48	LRI 1.33	CKD 1.09	DM 1.02	EMF -0.13	IV -0.15	Self-harm -0.34	RI -0.51	Drowning -0.64
High-middle SDI	IHD 9.65	Stroke 9.10	COPD 4.62	LRI 2.10	HHD 1.07	DM 1.05	CKD 0.88	EMF -0.16	IV -0.29	RI -0.66	Drowning -0.67	Self-harm -0.70
High SDI	IHD 5.00	COPD 3.78	Stroke 3.46	LRI 2.92	CKD 1.11	DM 0.10	HHD 0.55	EMF -0.08	IV -0.13	Drowning -0.33	RI -0.47	Self-harm -0.74

**High income**												
Australasia	IHD 4.07	COPD 2.35	Stroke 1.82	LRI 0.75	CKD 0.74	DM 0.61	HHD 0.18	EMF -0.04	IV -0.06	Drowning -0.17	RI -0.19	Self-harm -0.55
Western Europe	IHD 4.51	COPD 4.03	Stroke 3.86	LRI 2.92	DM 1.09	CKD 0.99	HHD 0.74	IV -0.05	EMF -0.07	Drowning -0.19	RI -0.31	Self-harm -0.58
High-income Asia Pacific	LRI 3.60	Stroke 3.28	IHD 2.37	COPD 1.47	CKD 0.86	DM 0.51	HHD 0.27	IV -0.03	EMF -0.06	RI -0.19	Drowning -0.56	Self-harm -0.75
High-income North America	IHD 7.09	COPD 5.51	Stroke 2.99	LRI 2.11	CKD 1.49	DM 1.34	HHD 0.72	EMF -0.10	IV -0.29	Drowning -0.30	RI -0.57	Self-harm -0.89
Southern Latin America	LRI 8.09	IHD 5.55	Stroke 5.24	COPD 4.91	CKD 2.28	DM 1.91	HHD 1.12	EMF -0.19	IV -0.41	Drowning -0.43	Self-harm -0.88	RI -1.02
**Central and eastern Europe, and central Asia**												
Central Asia	IHD 29.78	Stroke 18.07	COPD 6.35	LRI 5.66	DM 2.83	HHD 2.30	CKD 1.36	EMF -0.19	IV -0.41	Self-harm -1.09	RI -1.16	Drowning -1.37
Central Europe	Stroke 11.35	IHD 11.14	COPD 3.55	LRI 3.26	HHD 1.76	DM 1.61	CKD 0.83	IV -0.10	EMF -0.14	Drowning -0.50	RI -0.64	Self-harm -0.79
Eastern Europe	IHD 21.67	Stroke 15.78	COPD 2.90	LRI 2.64	DM 0.65	HHD 0.45	CKD 0.38	EMF -0.35	IV -1.36	Drowning -1.54	RI -1.73	Self-harm -2.72
**Latin America and Caribbean**												
Central Latin America	IHD 4.18	Stroke 2.25	COPD 2.15	LRI 2.12	DM 1.41	HHD 0.90	CKD 0.49	EMF -0.06	IV -0.16	Self-harm -0.19	RI -0.27	Drowning -0.66

Caribbean	IHD 1.59	Stroke 0.44	HHD 0.23	COPD 0.20	CKD 0.14	DM <0.01	LRI -0.01	EMF -0.02	RI -0.12	Self-harm -0.14	Drowning -0.25	IV -0.27
Tropical Latin America	IHD 2.30	COPD 1.18	Stroke 1.16	LRI 0.44	CKD 0.42	HHD 0.42	DM 0.40	EMF -0.02	RI -0.10	Self-harm -0.14	Drowning -0.22	IV -0.41
Andean Latin America	LRI 5.16	IHD 3.41	Stroke 2.48	CKD 2.01	COPD 1.69	DM 1.43	HHD 0.53	EMF -0.14	IV -0.23	Self-harm -0.25	Drowning -0.53	RI -0.70
**North Africa and Middle East**	IHD 15.75	Stroke 5.89	COPD 3.42	HHD 2.39	LRI 2.32	CKD 2.14	DM 1.98	IV -0.17	EMF -0.20	Self-harm -0.24	Drowning -0.30	RI -0.82
**South Asia**	IHD 5.84	COPD 4.35	Stroke 1.98	LRI 1.06	DM 0.79	CKD 0.77	HHD 0.43	EMF -0.04	IV -0.15	Drowning -0.35	Self-harm -0.43	RI -0.50
**Southeast Asia, east Asia, and Oceania**												
East Asia	Stroke 12.34	COPD 10.82	IHD 8.93	HHD 1.83	LRI 1.70	CKD 0.93	DM 0.87	IV -0.05	EMF -0.24	Self-harm -0.46	RI -0.79	Drowning -1.21
Southeast Asia	IHD 1.84	Stroke 1.62	COPD 0.77	HHD 0.37	CKD 0.30	DM 0.18	LRI 0.04	EMF -0.01	IV -0.04	Self-harm -0.10	RI -0.13	Drowning -0.25
Oceania	IHD 5.00	COPD 4.94	Stroke 2.72	DM 1.38	HHD 0.91	LRI 0.85	CKD 0.36	EMF -0.04	Self-harm -0.12	RI -0.17	IV -0.19	Drowning -0.39
**Sub-Saharan Africa**												
Southern Sub-Saharan Africa	IHD 6.37	Stroke 5.73	LRI 5.66	DM 4.74	COPD 3.99	HHD 2.80	CKD 2.20	EMF -0.15	RI -0.27	Self-harm -0.66	Drowning -0.72	IV -0.81
Eastern Sub-Saharan	IHD	Stroke	HHD	COPD	LRI	CKD	DM	EMF	RI	IV	Drowning	Self-harm

Africa	3.93	3.50	1.80	1.62	1.59	0.88	0.81	-0.09	-0.14	-0.18	-0.26	-0.35
Central Sub-Saharan Africa	IHD 3.58	Stroke 2.14	HHD 1.73	COPD 1.33	CKD 0.49	DM 0.26	EMF -0.04	LRI -0.06	IV -0.09	Drowning -0.24	RI -0.33	Self-harm -0.36
Western Sub-Saharan Africa	IHD 1.66	Stroke 0.79	LRI 0.69	CKD 0.37	COPD 0.33	HHD 0.22	DM 0.18	EMF -0.03	Drowning -0.07	Self-harm -0.13	IV -0.14	RI -0.29

≥ 10	5 to 10	3 to 5	1.5 to 3	0 to 1.5	-0.5 to 0	-1 to -0.5	≤ -1

Table S3. Twelve leading causes of total DALY rates attributable to low temperatures in 2019 by region. Causes are ranked according to global estimates of age-standardized DALY rates (per 100,000 people) provided by the GBD Study 2019. The rates are listed and colored in each cell: Shades of blue indicate DALY rates less than zero whereas red indicates death rates greater than zero. (Notes: DALY=disability-adjusted life years; IHD=Ischemic heart disease; COPD=Chronic obstructive pulmonary disease; LRI=Lower respiratory infections; CKD=Chronic kidney disease; DM=Diabetes mellitus; HHD=Hypertensive heart disease; EMF=Exposure to mechanical forces; IV=Interpersonal violence; RI=Road injuries.)

Region	***1***	***2***	***3***	***4***	***5***	***6***	***7***	***8***	***9***	***10***	***11***	***12***
**Global**	IHD 126.60	Stroke 103.92	COPD 74.43	LRI 53.73	CKD 20.43	DM 19.21	HHD 15.04	EMF -5.24	IV -9.96	Self-harm -21.16	RI -24.10	Drowning -26.97
**Social-demographic index**												
Low SDI	IHD 112.52	COPD 65.71	Stroke 61.22	LRI 59.96	CKD 21.99	HHD 19.63	DM 18.20	EMF -3.84	IV -11.20	Self-harm -13.79	Drowning -17.51	RI -19.48
Low-middle SDI	IHD 117.49	COPD 90.48	Stroke 81.25	LRI 43.38	CKD 19.67	DM 16.62	HHD 13.05	EMF -3.63	IV -8.81	Self-harm -19.95	Drowning -23.69	RI -23.80
Middle SDI	IHD 135.75	Stroke 123.25	COPD 90.44	LRI 40.40	CKD 22.81	HHD 21.67	DM 20.50	EMF -6.20	IV -8.39	Self-harm -13.58	RI -22.63	Drowning -33.13
High-middle SDI	IHD 158.05	Stroke 149.10	COPD 66.00	LRI 48.13	DM 18.95	CKD 15.70	HHD 14.02	EMF -7.43	IV -14.35	Self-harm -31.17	RI -32.04	Drowning -35.21
High SDI	IHD 84.68	COPD 59.90	Stroke 53.04	LRI 42.32	DM 19.39	CKD 17.86	HHD 8.92	EMF -3.67	IV -7.67	Drowning -14.56	RI -23.29	Self-harm -32.74

**High income**												
Australasia	IHD 60.72	COPD 35.44	Stroke 24.25	DM 10.54	CKD 10.49	LRI 10.25	HHD 2.35	EMF -1.80	IV -3.05	RI -9.50	Drowning -9.94	Self-harm -26.79
Western Europe	IHD 68.97	COPD 59.37	Stroke 53.70	LRI 40.10	DM 17.47	CKD 12.59	HHD 8.17	IV -2.37	EMF -2.71	Drowning -9.27	RI -15.19	Self-harm -24.85
High-income Asia Pacific	Stroke 52.12	LRI 48.51	IHD 38.36	COPD 19.52	CKD 12.61	DM 9.95	HHD 3.22	IV -1.34	EMF -2.41	RI -7.82	Drowning -16.37	Self-harm -32.34
High-income North America	IHD 122.19	COPD 90.75	Stroke 47.78	LRI 36.78	DM 28.62	CKD 26.18	HHD 15.20	EMF -4.72	IV -16.50	Drowning -17.23	RI -27.79	Self-harm -41.23
Southern Latin America	LRI 141.46	IHD 96.16	Stroke 93.92	COPD 77.24	CKD 40.21	DM 37.00	HHD 15.45	EMF -9.31	IV -22.52	Drowning -24.71	Self-harm -42.20	RI -48.53
**Central Europe, eastern Europe, and central Asia**												
Central Asia	IHD 516.00	Stroke 326.14	LRI 301.39	COPD 107.84	DM 68.51	HHD 37.19	CKD 36.87	EMF -9.01	IV -19.27	Self-harm -50.75	RI -55.36	Drowning -82.18
Central Europe	Stroke 178.61	IHD 173.22	LRI 83.31	COPD 62.17	DM 30.79	HHD 26.10	CKD 15.99	IV -4.51	EMF -6.41	Drowning -24.82	RI -31.03	Self-harm -33.93
Eastern Europe	IHD 377.73	Stroke 270.22	LRI 98.02	COPD 54.16	DM 14.76	CKD 9.21	HHD 8.00	EMF -16.61	IV -66.60	Drowning -81.86	RI -90.87	Self-harm -129.33
**Latin America and Caribbean**												
Central Latin America	IHD 73.29	CKD 52.46	DM 46.15	COPD 31.24	LRI 26.93	Stroke 26.14	HHD 7.24	EMF -3.03	RI -7.60	Self-harm -9.45	Drowning -15.40	IV -35.58

Caribbean	IHD 31.48	Stroke 9.35	HHD 4.39	CKD 3.62	COPD 3.58	DM -0.01	EMF -1.27	LRI -2.41	RI -5.91	Self-harm -6.10	IV -14.93	Drowning -16.83
Tropical Latin America	IHD 45.69	Stroke 21.45	COPD 19.58	LRI 9.01	CKD 8.59	DM 7.74	HHD 6.94	EMF -0.81	RI -4.79	Self-harm -6.52	Drowning -12.61	IV -22.95
Andean Latin America	LRI 119.93	IHD 57.47	Stroke 48.10	CKD 40.24	DM 29.71	COPD 23.68	HHD 8.38	EMF -6.84	IV -12.31	Self-harm -12.47	Drowning -29.17	RI -31.19
**North Africa and Middle East**	IHD 289.91	Stroke 100.86	LRI 70.72	COPD 54.30	CKD 40.47	DM 38.11	HHD 37.49	IV -9.20	EMF -9.91	Self-harm -11.14	Drowning -18.70	RI -37.93
**South Asia**	IHD 125.19	COPD 70.59	LRI 45.96	Stroke 40.04	CKD 19.75	DM 16.22	HHD 7.32	EMF -1.91	IV -7.49	Drowning -19.69	Self-harm -21.28	RI -21.95
**Southeast Asia, east Asia, and Oceania**												
East Asia	Stroke 208.46	COPD 146.63	IHD 138.50	LRI 44.94	HHD 24.73	CKD 18.66	DM 16.68	IV -2.81	EMF -11.56	Self-harm -16.94	RI -37.23	Drowning -67.04
Southeast Asia	IHD 34.70	Stroke 30.52	COPD 12.51	CKD 6.76	HHD 6.61	DM 3.74	LRI 2.14	EMF -0.77	IV -2.07	Self-harm -4.17	RI -5.76	Drowning -14.49
Oceania	IHD 113.31	COPD 91.32	Stroke 60.93	LRI 47.00	DM 33.86	HHD 19.41	CKD 10.12	EMF -2.15	Self-harm -5.31	RI -8.47	IV -9.86	Drowning -25.53
**Sub-Saharan Africa**												
Southern Sub-Saharan Africa	LRI 168.72	IHD 114.71	Stroke 98.92	DM 92.31	COPD 67.63	HHD 46.39	CKD 46.29	EMF -7.27	RI -12.94	Self-harm -30.15	Drowning -41.38	IV -43.30
Eastern Sub-Saharan	IHD	Stroke	LRI	HHD	COPD	CKD	DM	EMF	RI	IV	Self-harm	Drowning

Africa	71.87	66.24	39.94	30.87	28.55	18.14	16.19	-3.14	-5.38	-8.69	-11.81	-13.40
Central Sub-Saharan	IHD	Stroke	HHD	COPD	CKD	DM	LRI	EMF	IV	Self-harm	Drowning	RI
Africa	67.85	41.35	30.96	22.62	10.92	5.57	-1.59	-1.60	-4.73	-12.37	-13.79	-16.00
Western Sub-Saharan	LRI	IHD	Stroke	CKD	COPD	HHD	DM	EMF	Self-harm	Drowning	IV	RI
Africa	36.82	29.65	15.48	8.52	5.95	4.31	3.75	-1.47	-4.30	-4.31	-7.10	-12.55

≥ 250	100 to 250	50 to 100	25 to 50	0 to 25	-20 to 0	-40 to -20	≤ -40

Table S4. Global age-standardized rates of death and DALY attributable to high temperature from 1990-2019.

	DALY				Death			
Age-standardized rate per 100000 people	1990	2005	2019	Temporal trend from 1990 to 2019(β)	1990	2005	2019	Temporal trend from 1990 to 2019(β)
Region								
**Social-demographic index**								
Low SDI	492.90 (310.20, 704.85)	373.51 (236.42, 524.49)	293.39 (270.23, 391.40)	-8.33*	20.39 (14.11, 27.03)	17.79 (12.48, 22.93)	15.56 (10.91, 20.07)	-0.23*
Low-middle SDI	524.15 (360.21, 702.87)	378.90 (262.14, 524.49)	302.07 (209.58, 397.88)	-8.10*	25.42 (19.12, 31.76)	21.58 (16.34, 26.70)	17.92 (13.61, 22.31)	-0.31*
Middle SDI	780.71 (662.88, 906.59)	516.37 (236.42, 524.49)	370.89 (312.07, 432.67)	-13.83*	42.27 (37.15, 48.38)	33.23 29.18, 37.41)	24.55 (20.91, 28.27)	-0.69*
High-middle SDI	799.99 (661.01, 964.91)	557.12 (236.42, 524.49)	349.75 (278.75, 429.59)	-17.00*	49.63 (41.29, 58.57)	39.36 (32.29, 47.10)	25.99 (21.38, 31.07)	-0.97*
High SDI	407.89 (308.84, 511.94)	266.50 (211.37, 324.77)	204.18 (165.59, 245.73)	-7.33*	30.59 (37.14, 24.02)	20.95 (17.14, 24.75)	16.04 (13.09, 18.92)	-0.54*
**High income**								

Australasia	292.67 (235.28, 351.13)	144.85 (115.97, 173.53)	102.95 (80.46, 123.73)	-6.34*	21.95 (18.21, 25.69)	12.67 (10.53, 14.79)	9.51 (7.79, 11.07)	-0.44*
Western Europe	445.69 (319.52, 575.06)	279.20 (319.52, 575.06)	205.99 (160.63, 252.19)	-8.16*	33.59 (25.64, 41.66)	22.43 (17.73, 29.96)	16.94 (13.59, 20.28)	-0.60*
High-income Asia Pacific	356.19 (291.42, 423.77)	186.26 (143.30, 230.82)	124.00 (90.36, 156.53)	-7.32*	27.16 (23.37, 31.14)	15.56 (12.73, 18.17)	10.77 (12.73, 18.17)	-0.55*
High-income North America	368.92 (287.71, 450.09)	304.75 (249.38, 364.22)	260.03 (213.84, 307.54)	-5.64*	27.30 (22.46, 32.44)	22.64 (19.09, 26.39)	19.12 (16.08, 22.15)	-0.41*
Southern Latin America	590.33 (468.54, 731.64)	427.76 (343.72 532.98)	354.15 (283.00, 441.70)	-7.71*	36.78 (30.33, 44.29)	30.75 (25.77, 36.85)	26.17 (21.67, 31.37)	-0.35*
**Central Europe, eastern Europe, and central Asia**								
Central Asia	1603.1 (1298.1, 1997.6)	1701.7 (1403.0, 2039.5)	1177.4 (939.07, 1442.9)	-26.00*	61.69 (50.22, 74.35)	80.64 (65.00, 97.05)	62.12 (49.55, 76.10)	-0.24
Central Europe	1002.00 (758.77, 1265.53)	732.74 (551.15, 905.65)	469.47 (342.75, 599.60)	-20.51*	56.50 (42.58, 71.72)	45.95 (34.90, 57.10)	31.35 (23.55, 39.54)	-1.03*
Eastern Europe	556.37 (283.24,	668.53 (304.05,	446.82 (226.57,	-4.95	50.80 (33.69,	55.57 (36.22,	36.77 (24.29,	-0.76*

	906.87)	1123.76)	711.57)		72.66)	81.88)	52.99)	
**Latin America and Caribbean**								
Central Latin America	328.23 (265.30, 394.09)	232.48 (191.16, 876.84)	192.40 (151.75, 239.47)	-4.23*	18.20 (15.35, 21.13)	14.00 (11.87, 16.33)	12.16 (9.86, 14.78)	-0.20*
Caribbean	28.41 (-0.54, 72.52)	29.24 (2.05, 64.32)	4.96 (-17.28, 27.37)	-0.71	3.18 (1.49, 5.62)	3.31 (1.88, 5.22)	1.78 (0.90, 3.04)	-0.04
Tropical Latin America	232.49 (152.09, 326.10)	114.85 (75.62, 163.73)	71.33 (45.70, 102.53)	-4.70*	14.09 (10.03, 18.83)	8.06 (5.77, 10.66)	5.44 (3.88, 7.26)	-0.26*
Andean Latin America	757.44 (567.25, 976.43)	363.26 (273.74, 456.96)	235.53 (168.31, 315.94)	-13.08*	28.82 (23.58, 35.10)	19.12 (15.58, 23.10)	14.86 (11.24, 18.98)	-0.38*
**North Africa and Middle East**	929.55 (751.05, 1117.87)	656.80 (528.51, 777.40)	544.98 (432.34, 657.08)	-14.85*	44.29 (36.48, 51.63)	35.07 (28.58, 40.86)	32.17 (25.87, 38.01)	-0.49*
**South Asia**	348.96 (130.78, 595.78)	296.69 (131.59, 479.31)	252.76 (121.98, 397.76)	-4.22	17.04 (7.98, 26.35)	15.94 (8.12, 23.32)	13.74 (19.82, 7.38)	-0.18
**Southeast Asia, east Asia, and Oceania**								
East Asia	1278.8 (1054.2,	721.58 (613.16,	463.03 (380.55,	-26.85*	73.01 (62.29,	51.43 (44.57,	34.69 (29.11,	-1.41*

	1504.2)	830.32)	558.21)		83.49)	57.95)	41.11)	
Southeast Asia	135.90 (72.75, 217.12)	108.47 (60.48, 162.23)	69.72 (39.26, 106.27)	-2.02*	7.99 (5.01, 11.35)	7.01 (4.39, 9.82)	4.59 (2.81, 6.69)	-0.10*
Oceania	462.69 (346.31, 600.63)	445.65 (323.02, 599.41)	324.64 (226.43, 446.54)	-3.44*	19.39 (15.00, 24.33)	19.90 (15.12, 25.34)	15.26 (11.43, 19.82)	-0.09
**Sub-Saharan Africa**								
Southern Sub-Saharan Africa	706.48 (571.08, 872.30)	801.46 (666.01, 959.12)	499.93 (405.09, 619.02)	-5.59$^{\#}$	32.67 (27.79, 38.03)	39.52 (34.37, 45.39)	28.87 (25.04, 33.39)	-0.08
Eastern Sub-Saharan Africa	558.34 (367.80, 783.96)	342.10 (227.71, 479.36)	229.39 (155.42, 319.19)	-11.26$^{\#}$	22.17 (16.31, 29.19)	16.54 (12.26, 21.72)	13.11 (9.46, 17.20)	-0.32*
Central Sub-Saharan Africa	286.10 (120.78, 508.75)	205.36 (84.95, 365.87)	129.19 (84.95, 365.87)	-5.84*	14.43 (9.08, 21.38)	12.08 (7.47, 17.19)	8.41 (4.87, 12.59)	-0.21*
Western Sub-Saharan Africa	123.88 (-59.00, 337.71)	92.60 (-40.70, 248.78)	74.76 (-34.79, 207.10)	-2.84	4.80 (0.73, 9.54)	3.96 (0.61, 7.93)	3.57 (0.55, 7.28)	-0.07
	DALY				Death			
Age-standardized rate per 100000 people	1990	2005	2019	Temporal trend from 1990 to 2019(β)	1990	2005	2019	Temporal trend from 1990 to 2019(β)

Region								
**Social-demographic index**								
Low SDI	492.90 (310.20, 704.85)	373.51 (236.42, 524.49)	293.39 (270.23, 391.40)	-8.70*	20.39 (14.11, 27.03)	17.79 (12.48, 22.93)	15.56 (10.91, 20.07)	-0.68*
Low-middle SDI	524.15 (360.21, 702.87)	378.90 (262.14, 524.49)	302.07 (209.58, 397.88)	-8.68*	25.42 (19.12, 31.76)	21.58 (16.34, 26.70)	17.92 (13.61, 22.31)	-0.23*
Middle SDI	780.71 (662.88, 906.59)	516.37 (236.42, 524.49)	370.89 (312.07, 432.67)	-14.96*	42.27 (37.15, 48.38)	33.23 29.18, 37.41)	24.55 (20.91, 28.27)	-0.32*
High-middle SDI	799.99 (661.01, 964.91)	557.12 (236.42, 524.49)	349.75 (278.75, 429.59)	-17.70*	49.63 (41.29, 58.57)	39.36 (32.29, 47.10)	25.99 (21.38, 31.07)	-0.70*
High SDI	407.89 (308.84, 511.94)	266.50 (211.37, 324.77)	204.18 (165.59, 245.73)	-8.19*	30.59 (37.14, 24.02)	20.95 (17.14, 24.75)	16.04 (13.09, 18.92)	-0.98*
**High income**								
Australasia	292.67 (235.28, 351.13)	144.85 (115.97, 173.53)	102.95 (80.46, 123.73)	-7.18*	21.95 (18.21, 25.69)	12.67 (10.53, 14.79)	9.51 (7.79, 11.07)	-0.48*
Western Europe	445.69 (319.52, 575.06)	279.20 (319.52, 575.06)	205.99 (160.63, 252.19)	-9.36*	33.59 (25.64, 41.66)	22.43 (17.73, 29.96)	16.94 (13.59, 20.28)	-0.66*

High-income Asia Pacific	356.19 (291.42, 423.77)	186.26 (143.30, 230.82)	124.00 (90.36, 156.53)	-8.25*	27.16 (23.37, 31.14)	15.56 (12.73, 18.17)	10.77 (12.73, 18.17)	-0.61*
High-income North America	368.92 (287.71, 450.09)	304.75 (249.38, 364.22)	260.03 (213.84, 307.54)	-5.68*	27.30 (22.46, 32.44)	22.64 (19.09, 26.39)	19.12 (16.08, 22.15)	-0.41*
Southern Latin America	590.33 (468.54, 731.64)	427.76 (343.72 532.98)	354.15 (283.00, 441.70)	-8.11*	36.78 (30.33, 44.29)	30.75 (25.77, 36.85)	26.17 (21.67, 31.37)	-0.36*
**Central Europe, eastern Europe, and central Asia**								
Central Asia	1603.1 (1298.1, 1997.6)	1701.7 (1403.0, 2039.5)	1177.4 (939.07, 1442.9)	-24.63*	61.69 (50.22, 74.35)	80.64 (65.00, 97.05)	62.12 (49.55, 76.10)	-0.29#
Central Europe	1002.00 (758.77, 1265.53)	732.74 (551.15, 905.65)	469.47 (342.75, 599.60)	-21.57*	56.50 (42.58, 71.72)	45.95 (34.90, 57.10)	31.35 (23.55, 39.54)	-1.05*
Eastern Europe	556.37 (283.24, 906.87)	668.53 (304.05, 1123.76)	446.82 (226.57, 711.57)	-4.50#	50.80 (33.69, 72.66)	55.57 (36.22, 81.88)	36.77 (24.29, 52.99)	-0.75*
**Latin America and Caribbean**								
Central Latin America	328.23 (265.30, 394.09)	232.48 (191.16, 876.84)	192.40 (151.75, 239.47)	-4.45*	18.20 (15.35, 21.13)	14.00 (11.87, 16.33)	12.16 (9.86, 14.78)	-0.21*

Caribbean	28.41 (-0.54, 72.52)	29.24 (2.05, 64.32)	4.96 (-17.28, 27.37)	-0.72*	3.18 (1.49, 5.62)	3.31 (1.88, 5.22)	1.78 (0.90, 3.04)	-0.04*
Tropical Latin America	232.49 (152.09, 326.10)	114.85 (75.62, 163.73)	71.33 (45.70, 102.53)	-5.29*	14.09 (10.03, 18.83)	8.06 (5.77, 10.66)	5.44 (3.88, 7.26)	-0.28*
Andean Latin America	757.44 (567.25, 976.43)	363.26 (273.74, 456.96)	235.53 (168.31, 315.94)	-15.47*	28.82 (23.58, 35.10)	19.12 (15.58, 23.10)	14.86 (11.24, 18.98)	-0.39*
**North Africa and Middle East**	929.55 (751.05, 1117.87)	656.80 (528.51, 777.40)	544.98 (432.34, 657.08)	-15.99*	44.29 (36.48, 51.63)	35.07 (28.58, 40.86)	32.17 (25.87, 38.01)	-0.52*
**South Asia**	348.96 (130.78, 595.78)	296.69 (131.59, 479.31)	252.76 (121.98, 397.76)	-4.33*	17.04 (7.98, 26.35)	15.94 (8.12, 23.32)	13.74 (19.82, 7.38)	-0.18*
**Southeast Asia, east Asia, and Oceania**								
East Asia	1278.8 (1054.2, 1504.2)	721.58 (613.16, 830.32)	463.03 (380.55, 558.21)	-30.53*	73.01 (62.29, 83.49)	51.43 (44.57, 57.95)	34.69 (29.11, 41.11)	-1.45*
Southeast Asia	135.90 (72.75, 217.12)	108.47 (60.48, 162.23)	69.72 (39.26, 106.27)	-1.99*	7.99 (5.01, 11.35)	7.01 (4.39, 9.82)	4.59 (2.81, 6.69)	-0.10*
Oceania	462.69 (346.31,	445.65 (323.02,	324.64 (226.43,	-3.31*	19.39 (15.00,	19.90 (15.12,	15.26 (11.43,	-0.09*

	600.63)	599.41)	446.54)		24.33)	25.34)	19.82)	
**Sub-Saharan Africa**								
Southern Sub-Saharan Africa	706.48 (571.08, 872.30)	801.46 (666.01, 959.12)	499.93 (405.09, 619.02)	-4.97#	32.67 (27.79, 38.03)	39.52 (34.37, 45.39)	28.87 (25.04, 33.39)	-0.07
Eastern Sub-Saharan Africa	558.34 (367.80, 783.96)	342.10 (227.71, 479.36)	229.39 (155.42, 319.19)	-11.91*	22.17 (16.31, 29.19)	16.54 (12.26, 21.72)	13.11 (9.46, 17.20)	-0.32*
Central Sub-Saharan Africa	286.10 (120.78, 508.75)	205.36 (84.95, 365.87)	129.19 (84.95, 365.87)	-5.61*	14.43 (9.08, 21.38)	12.08 (7.47, 17.19)	8.41 (4.87, 12.59)	-0.21*
Western Sub-Saharan Africa	123.88 (-59.00, 337.71)	92.60 (-40.70, 248.78)	74.76 (-34.79, 207.10)	-3.18*	4.80 (0.73, 9.54)	3.96 (0.61, 7.93)	3.57 (0.55, 7.28)	-0.08*

Notes: # <0.05; *<0.001.

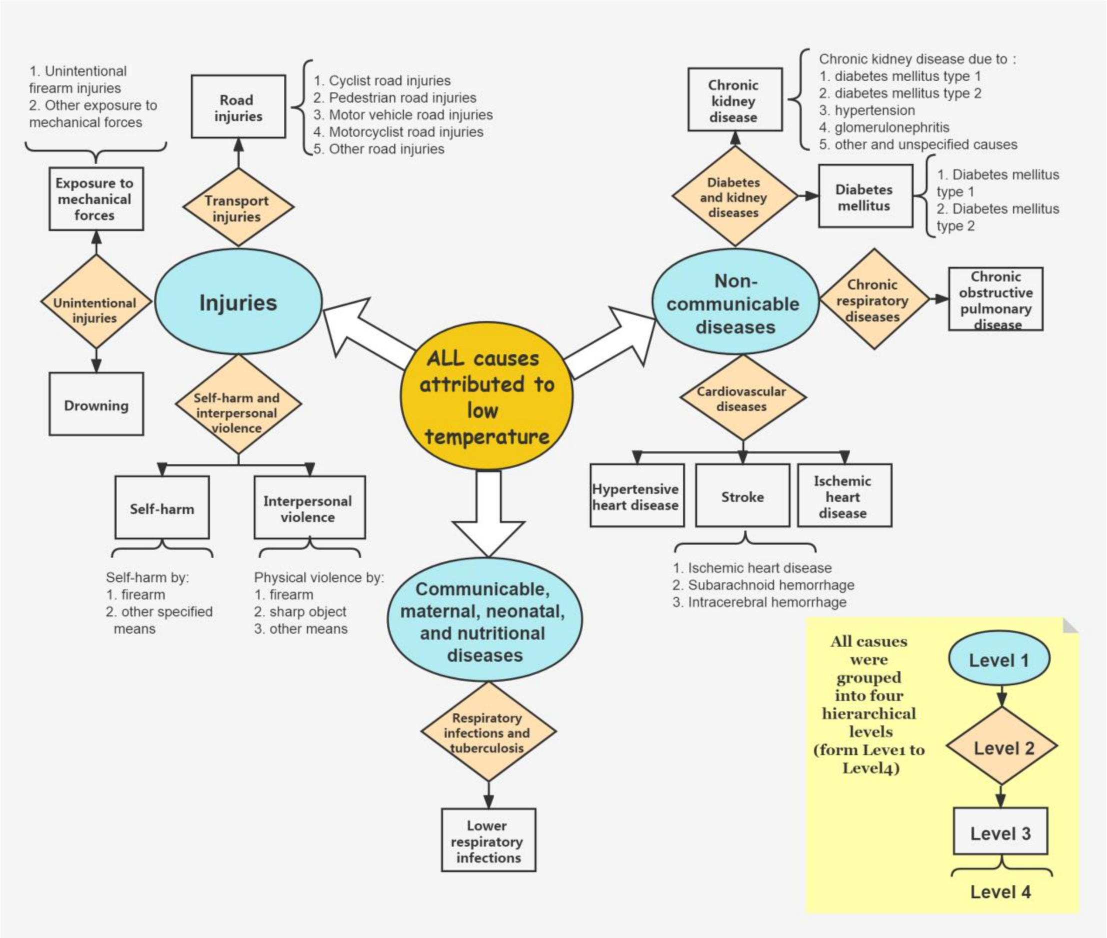

Figure S1. Low-temperature-related diseases studied within GBD 2019.

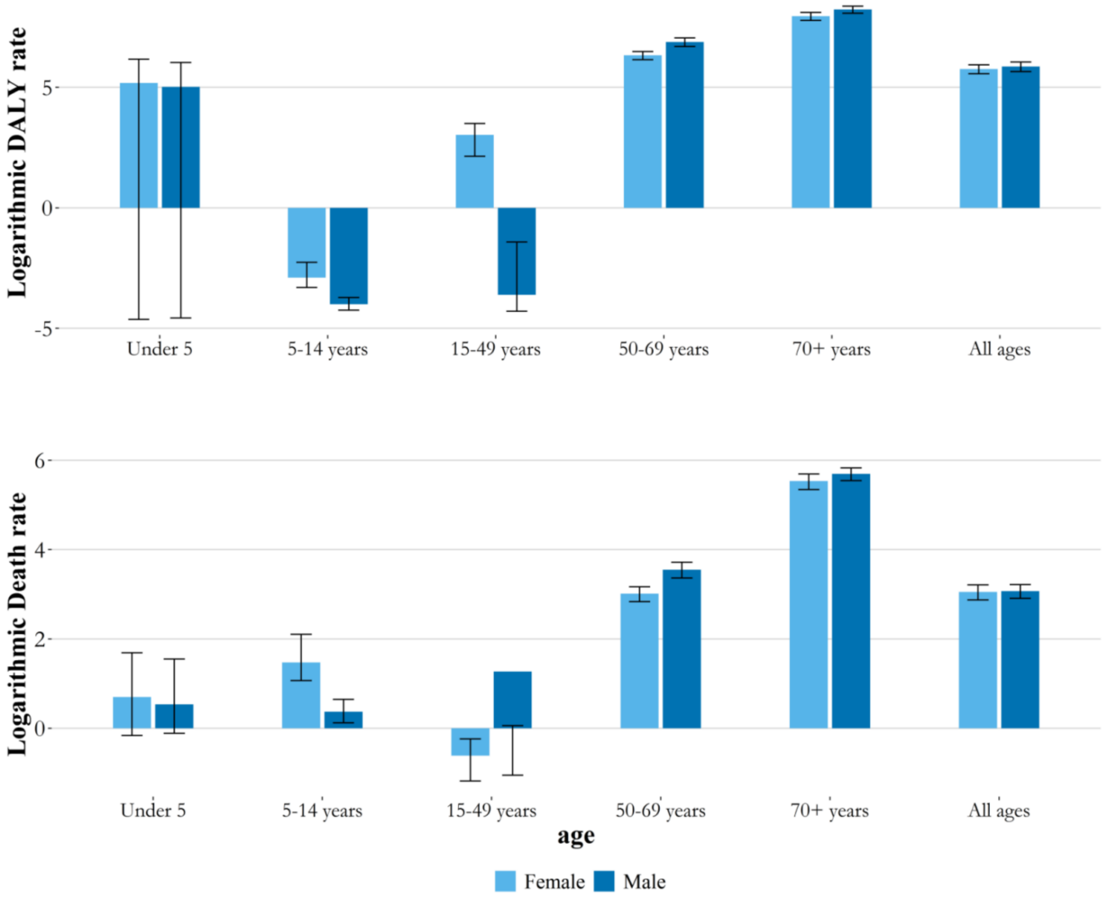

Figure S2. Age-standardized rates of deaths and DALYs attributable to low temperatures stratified by age and gender. DALY=disability-adjusted life year.

(A) Number of DALYs (Units: one thousand people)

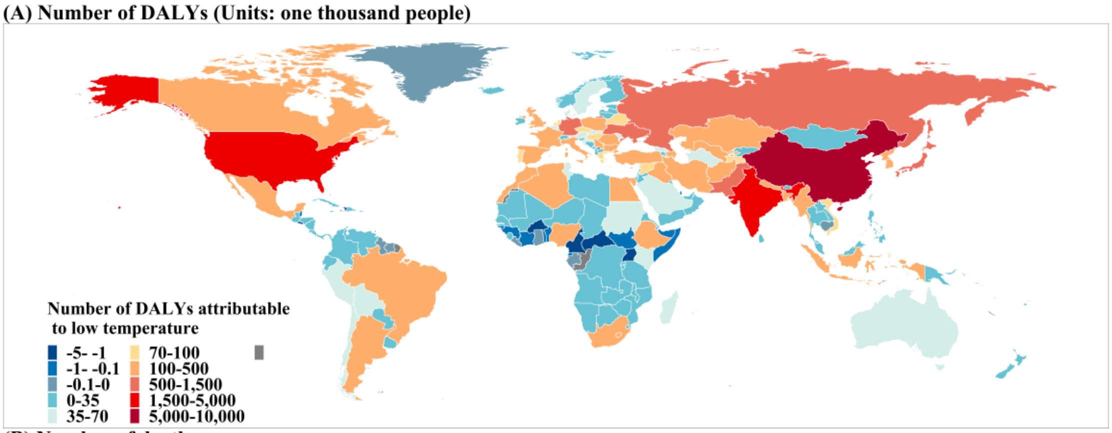

(B) Number of deaths

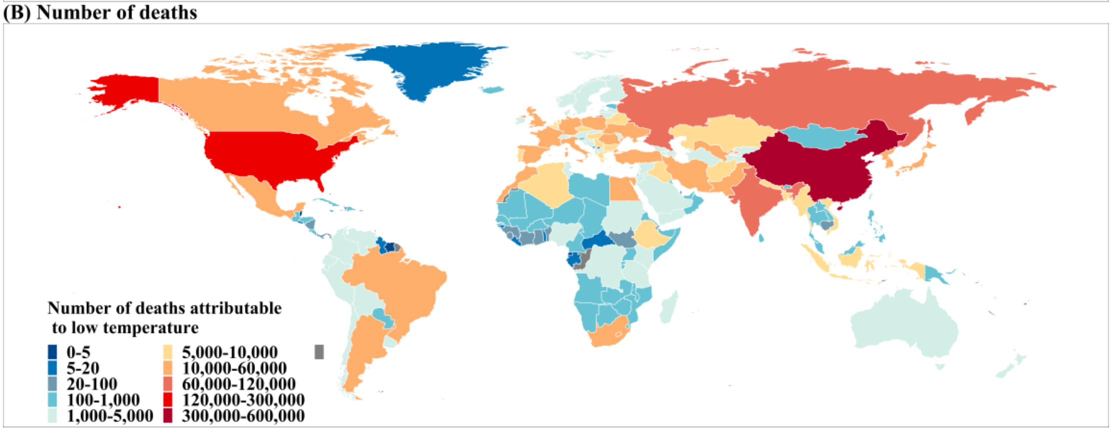

Figure S3. The number of low temperature-related deaths and DALYs across countries. DALYs=disability-adjusted life-years.

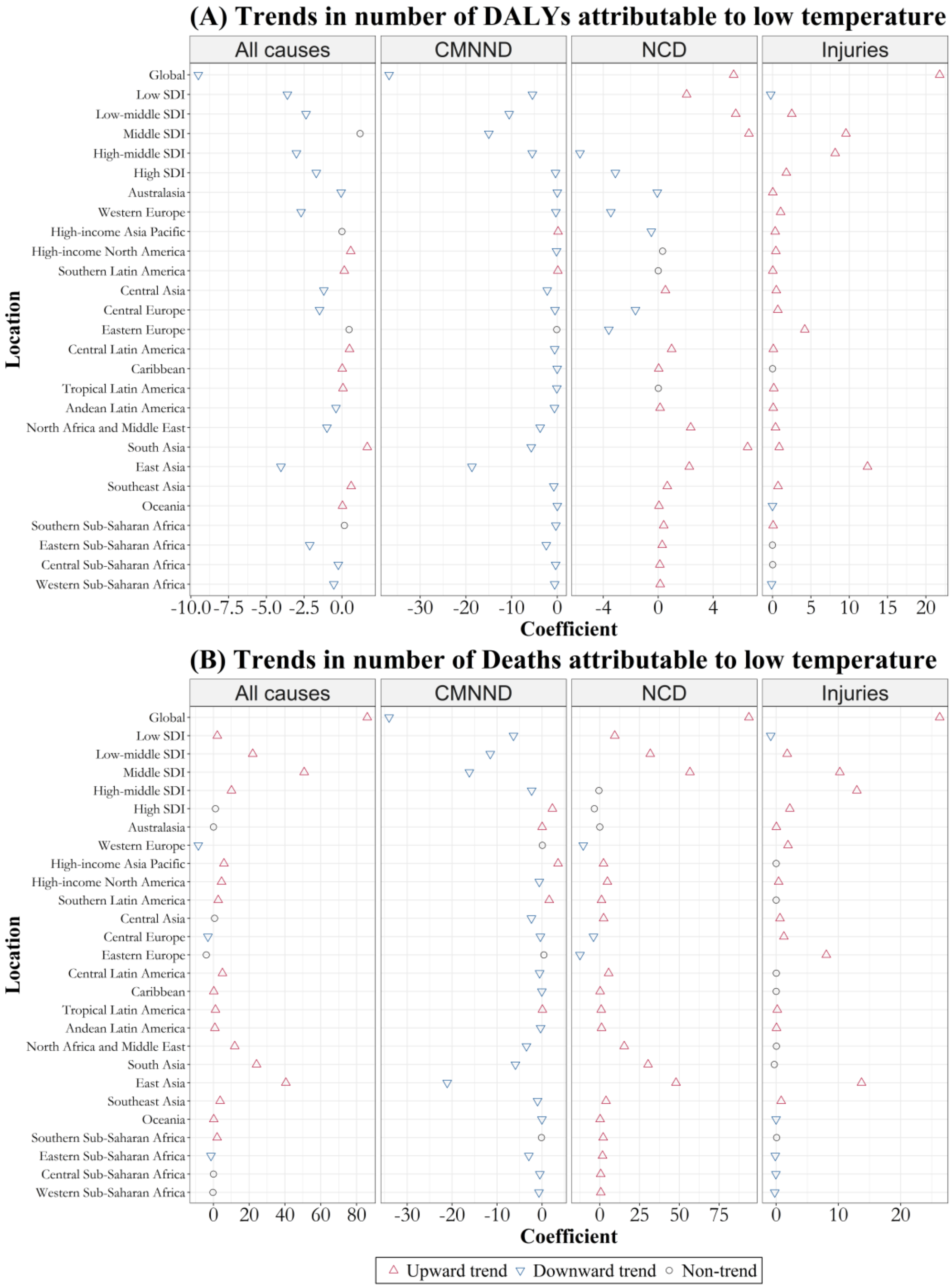

Figure S4. Temporal trends in numbers of DALYs and deaths attributable to low temperatures from 1990 to 2019 among 21 geographical and five SDI level regions. DALYs=disability-adjusted life-years; CMNND=communicable, maternal, neonatal, and nutritional diseases; NCD=Non-communicable diseases.

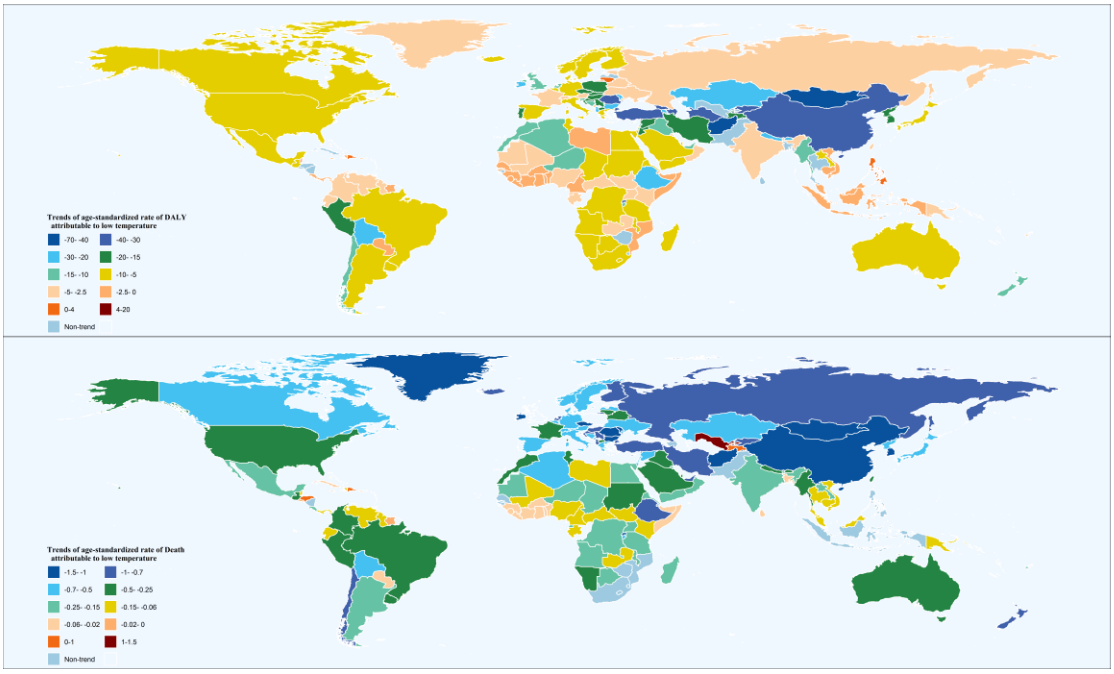

Figure S5. Temporal trends of age-standardized DALY and death rates attributable to low temperature across countries from 1990-2019 (the number referred to β value). DALY=disability-adjusted life year.

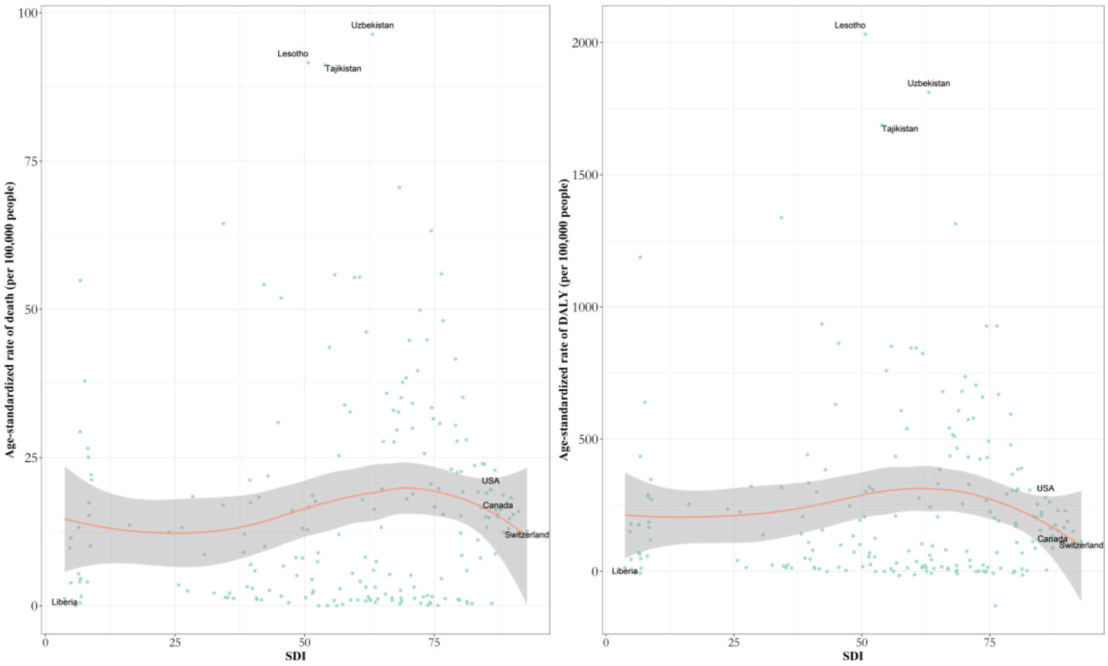

Figure S6. Correlation between SDI value and age-standardized rate of DALY and death attributable to low temperature by country. DALY=disability-adjusted life year.
